# Gene-Gene Combination Effect and Interactions among *ABCA1*, *APOA1*, *SR-B1*, and *CETP* Polymorphisms for Serum High-Density Lipoprotein-Cholesterol in the Japanese Population

**DOI:** 10.1371/journal.pone.0082046

**Published:** 2013-12-20

**Authors:** Akihiko Nakamura, Hideshi Niimura, Kazuyo Kuwabara, Toshiro Takezaki, Emi Morita, Kenji Wakai, Nobuyuki Hamajima, Yuichiro Nishida, Tanvir Chowdhury Turin, Sadao Suzuki, Keizo Ohnaka, Hirokazu Uemura, Etsuko Ozaki, Satoyo Hosono, Haruo Mikami, Michiaki Kubo, Hideo Tanaka

**Affiliations:** 1 Department of International Island and Community Medicine, Kagoshima University Graduate School of Medical and Dental Sciences, Kagoshima, Japan; 2 Education Center for Doctors in Remote Islands and Rural Areas, Kagoshima University Graduate School of Medical and Dental Sciences, Kagoshima, Japan; 3 Department of Preventive Medicine, Nagoya University Graduate School of Medicine, Nagoya, Japan; 4 Department of Healthcare Administration, Nagoya University Graduate School of Medicine, Nagoya, Japan; 5 Department of Preventive Medicine, Faculty of Medicine, Saga University, Saga, Japan; 6 Department of Health Science, Shiga University of Medical Science Otsu, Japan; 7 Department of Medicine, University of Calgary, Calgary, Canada; 8 Department of Public Health, Nagoya City University Graduate School of Medical Sciences, Nagoya, Japan; 9 Department of Geriatric Medicine, Graduate School of Medical Sciences, Kyushu University, Fukuoka, Japan; 10 Department of Preventive Medicine, Institute of Health Biosciences, University of Tokushima Graduate School, Tokushima, Japan; 11 Department of Epidemiology for Community Health and Medicine, Kyoto Prefectural University of Medicine, Kyoto, Japan; 12 Division of Epidemiology and Prevention, Aichi Cancer Center Research Institute, Nagoya, Japan; 13 Division of Cancer Registry, Prevention and Epidemiology, Chiba Cancer Center, Chiba, Japan; 14 Laboratory for Genotyping Development, Center for Genomic Medicine, RIKEN, Yokohama, Japan; University of Milan, Italy

## Abstract

**Background/Objective:**

Gene-gene interactions in the reverse cholesterol transport system for high-density lipoprotein-cholesterol (HDL-C) are poorly understood. The present study observed gene-gene combination effect and interactions between single nucleotide polymorphisms (SNPs) in *ABCA1*, *APOA1*, *SR-B1*, and *CETP* in serum HDL-C from a cross-sectional study in the Japanese population.

**Methods:**

The study population comprised 1,535 men and 1,515 women aged 35–69 years who were enrolled in the Japan Multi-Institutional Collaborative Cohort (J-MICC) Study. We selected 13 SNPs in the *ABCA1*, *APOA1*, *CETP*, and *SR-B1* genes in the reverse cholesterol transport system. The effects of genetic and environmental factors were assessed using general linear and logistic regression models after adjusting for age, sex, and region.

**Principal Findings:**

Alcohol consumption and daily activity were positively associated with HDL-C levels, whereas smoking had a negative relationship. The T allele of *CETP*, rs3764261, was correlated with higher HDL-C levels and had the highest coefficient (2.93 mg/dL/allele) among the 13 SNPs, which was statistically significant after applying the Bonferroni correction (*p*<0.001). Gene-gene combination analysis revealed that *CETP* rs3764261 was associated with high HDL-C levels with any combination of SNPs from *ABCA1*, *APOA1*, and *SR-B1*, although no gene-gene interaction was apparent. An increasing trend for serum HDL-C was also observed with an increasing number of alleles (*p*<0.001).

**Conclusions:**

The present study identified a multiplier effect from a polymorphism in *CETP* with *ABCA1*, *APOA1*, and *SR-B1*, as well as a dose-dependence according to the number of alleles present.

## Introduction

Coronary heart disease and cerebrovascular disease are the leading causes of mortality in both high- and low-income countries [1], [2]. A low level of high-density lipoprotein-cholesterol (HDL-C) is an important risk factor for these diseases [3], [4]. Environmental and genetic factors influence HDL-C levels; however, no specific treatments are available. A recent Mendelian randomization study reported that a genetic mechanism for raising serum HDL-C did not seem to lower the risk of myocardial infarction [5]. Further study is still required.

Several environmental factors have been reported to affect HDL-C levels. Alcohol consumption, habitual exercise, and high consumption of eggs and fish are positively associated with HDL-C levels [6]–[9]. Smoking leads to decreased levels of HDL-C [10]. Genetic factors, with or without environmental influence, also affect HDL-C levels. Several gene polymorphisms, especially in the genes encoding enzymes involved in the reverse cholesterol transport (RCT) system, have been reported to be associated with HDL-C levels [11]–[17]. The interaction between genetic and environmental factors has been investigated, but the reports have shown inconsistent results [18]–[20]. Furthermore, gene-gene interactions in the RCT system may modulate HDL-C levels, but few studies have been able clarify this effect, with the exception of one report from among the USA population [21].

HDL-C exerts an anti-atherogenic effect through several mechanisms such as anti-inflammation, anti-oxidation of low-density lipoprotein-cholesterol (LDL-C), and inhibition of vascular endothelial cell apoptosis. The RCT system also plays an important role in these processes [22]. The RCT system is involved in the transportation of cholesterol from the peripheral tissues to the liver, where the cholesterol is secreted into bile. ATP-binding-cassette A1 (ABCA1), apolipoprotein A-1 (ApoA-1), lecithin cholesterol acyltransferase (LCAT), cholesteryl ester transfer protein (CETP), and scavenger receptor class B1 (SR-B1) play important roles in the RCT system.

We are currently conducting the Japan Multi-Institutional Collaborative Cohort (J-MICC) study by compiling data from a relatively large number of subjects recruited from the Japanese population together with environmental data and DNA samples. To clarify the effects of gene-gene combination and interactions on HDL-C levels, we conducted a cross-sectional study by using data from the J-MICC study and focusing on gene polymorphisms in *ABCA1*, *APOA1*, *CETP*, and *SR-B1* in the RCT system.

## Subjects and Methods

### Ethics Statement

All participants provided written informed consent. The ethics committees of all participating institutes and universities approved the protocol. All data and samples were sent to the Nagoya University School of Medicine as linkable anonymizing data and de-identified samples. New identification numbers were then given for the combined dataset, which was linked to the analyzed data of gene polymorphisms. These data were sent to each collaborator without identifying lists.

The participating institutes and universities included: (1) Department of Preventive Medicine, Nagoya University Graduate School of Medicine, Nagoya, Japan; (2) Department of International Island and Community Medicine, Kagoshima University Graduate School of Medical and Dental Sciences, Kagoshima, Japan; (3) Department of Preventive Medicine, Faculty of Medicine, Saga University, Saga, Japan; (4) Department of Health Science, Shiga University of Medical Science, Otsu, Japan; (5) Department of Public Health, Nagoya City University Graduate School of Medical Sciences, Nagoya, Japan; (6) Department of Geriatric Medicine, Graduate School of Medical Sciences, Kyushu University, Fukuoka, Japan; (7) Department of Preventive Medicine, Institute of Health Biosciences, University of Tokushima Graduate School, Tokushima, Japan; (8) Department of Epidemiology for Community Health and Medicine, Kyoto Prefectural University of Medicine, Kyoto, Japan; (9) Division of Epidemiology and Prevention, Aichi Cancer Center Research Institute, Nagoya, Japan; (10) Division of Cancer Registry, Prevention and Epidemiology, Chiba Cancer Center, Chiba, Japan; and (11) Laboratory for Genotyping Development, Center for Genomic Medicine, RIKEN, Yokohama, Japan.

### Study Population

**Figure 1:**
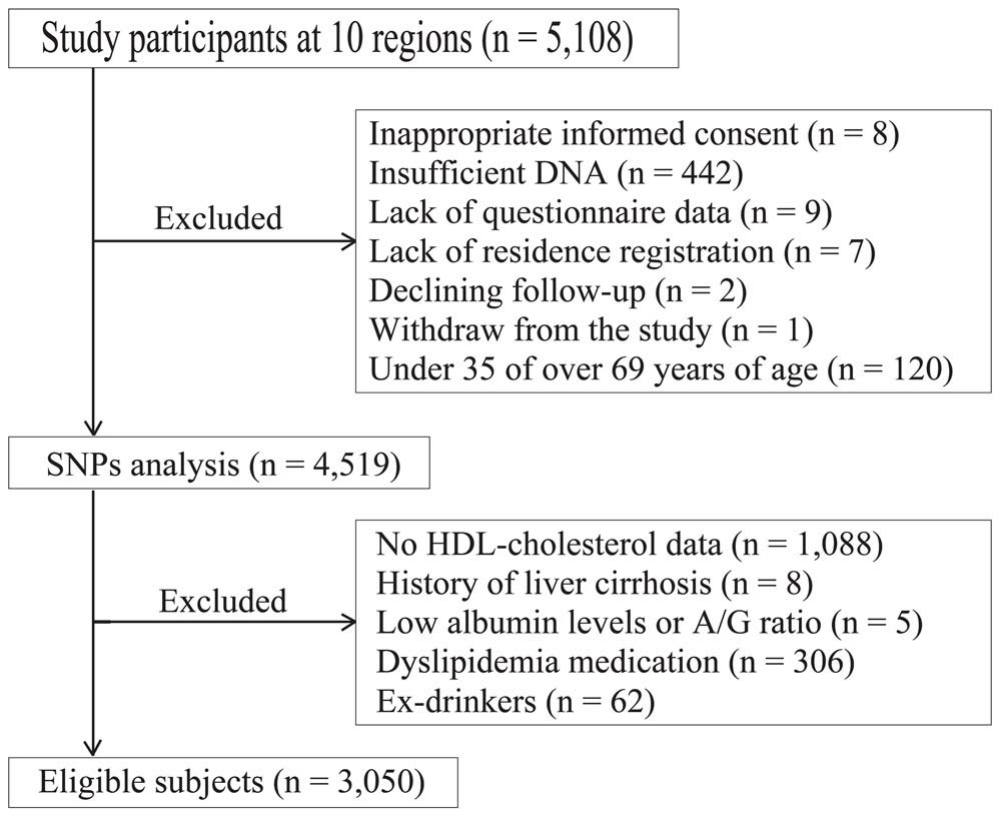
Diagram to list individuals who were excluded from the study sample.

**Figure 2:**
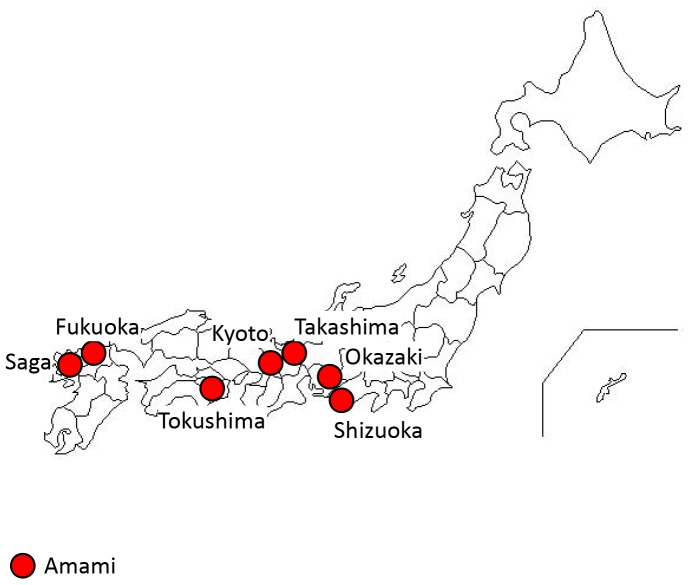
Location of the 8 study regions.

The J-MICC study has been conducted in 10 regions of Japan by 10 research institutes and universities since 2005, as described previously [23], [24]. In brief, the cohort participants were enrolled from the community through invitations mailed or leaflets distributed (3 regions) to patients on their first visit to a cancer hospital (1 region) or at health checkups (6 regions). First, we recruited 5,108 participants at the baseline of the J-MICC study (Figure 1). From these, we excluded the participants from whom we did not receive appropriate informed consent (n = 8), sufficient DNA (n = 442), questionnaire data (n = 9), or local government registration of residence in the study region (n = 7); anyone who had declined follow-up visits (n = 2); anyone who had withdrawn from the study (n = 1); and those who were under 35 or over 69 years of age (n = 120). Then, we proceeded with the SNPs analysis for the residual 4,519 subjects (2,124 men and 2,395 women). Furthermore, as the present study examined the association between HDL-C and SNPs, we also excluded those who had no HDL-C data (HDL-C examination was not included at one of the Cancer Center study region and at one of the community study regions; n = 1,088); those who had a history of liver cirrhosis (n = 8); those with low albumin levels as per blood examination (<3.5 g/dL); those with a low A/G ratio (<1.0; n = 5); those with a history of dyslipidemia with medication (n = 306); and those who had stopped drinking alcoholic beverages (excluded because most of them had stopped drinking owing to diseases associated with liver dysfunction; n = 62). Eventually, 3,050 subjects (1,535 men and 1,515 women) in 8 regions were deemed eligible for the present study. These regions were located in the western part of Japan, including the Amami Islands (Figure 2).

### Genotyping

First, we selected 5 genes encoding enzymes in the RCT system. Using these genes, we then selected 15 single nucleotide polymorphisms (SNPs) that have been reported to be associated with HDL-C levels in previous studies [11]–[17]: *ABCA1-565C>T* (rs2422493), R1587K (rs2230808), *-273G>C* (rs1800976), V771M (rs2066718), *-17C>G* (rs2740483), and V825I (rs2066715); *APOA1* A61T (rs12718465); *LCAT* (rs4986970); *CETP* Taq1B (rs708272), G/T (rs3764261), I405V (rs5882), and *-629C>A* (rs1800775); *SR-B1 A>G* (rs3782287), A350A (rs5888), and V135I (rs5891). The selected SNPs were genotyped by the multiplex polymerase chain reaction-based Invader assay at the Laboratory for Genotyping Development, Center for Genomic Medicine, RIKEN, as described previously [23]. Because no minor allele was found in the present study population, 1 SNP in *LCAT* (rs4986970) and 1 SNP in *SR-B1* (rs5891) were excluded from further analysis.

### Samples and Determination of Serum Lipid Levels

Venous blood samples were drawn during fasting. The mean duration of fasting was 9.8 hours and blood samples were drawn with the subjects in sitting position. The samples were separated into serum, plasma, and buffy coat, and were stored at −80°C on the day of sampling. Serum lipid levels were examined as part of the health checkup or for research purposes at the institutions affiliated with this study [25]. LDL-C values were estimated using the Friedewald formula when triglyceride levels were less than 400 mg/dL.

### Environmental Exposure

Information on environmental factors was obtained using a structural questionnaire [22]. The duration and intensity of daily activity (hard work and walking) and the frequency and intensity of habitual exercise were used to estimate the metabolic equivalents (METs). Alcohol consumption was estimated for 8 kinds of alcoholic beverages on the basis of intake frequency and ingested amount. Smoking habits were recorded as smoking status, number of cigarettes smoked, age at which he/she started smoking, and age at which he/she quit smoking. Dietary habits of the study participants were categorized into 8 groups on the basis of intake frequencies for each food item [26], [27].

### Statistical Analysis

Geographical differences in mean age and HDL-C values were confirmed by using one-way ANOVA between geographical regions. The characteristics of the clinical lipid profiles and the lifestyles of the study subjects were compared using a trend test and Chi-square test in 3 groups according to HDL-C levels, with almost an equal number of subjects in men and women.

The score variable (1–3) was used for all SNP analyses in the general linear model, because each SNP seemed to show a different effect on HDL-C levels depending on whether the additive or genotypic model was used. The coefficients of the genetic and environmental factors for the HDL-C value (the dependent variable) were estimated in the 2 models after adjusting for age (continuous), sex, smoking, drinking, daily activity (continuous METs), habitual exercise (continuous METs), intake of egg, meat, and fish, body mass index (BMI) and regions (site 1 to site 6). The Kyoto and Tokushima regions were combined into a single site because there was no difference in the distribution of SNPs between them, and the number of subjects in both regions was relatively small. We included each of the 6 regions in the model for adjustment variables except for the Amami Islands region, which was used as a reference site. METs per day were estimated from the information given in the questionnaire by using time and an intensity of 3.0 for walking and 4.5 for heavy work. Food intake was also estimated from the questionnaire on the basis of intake frequency; zero intake was included as the lowest category (“almost never”). To avoid any collinearity effect caused by gene linkage, 2 SNPs in *ABCA1* (rs2422493 and rs1800976), which were in close proximity, were not included as adjusted factors at the same time.

HDL-C levels were also compared according to each genotype by sex and SNP by using the trend test to assess whether the effect of each SNP was from the threshold or additive model.

Gene-gene combination effect and interaction was evaluated using a logistic regression model by using the dummy variable (0, 1) for SNPs to compare the magnitude of their odd ratios (ORs). From each gene, a representative SNP was selected that showed the lowest p-value and highest coefficient in the general linear model. We evaluated gene-gene combination effects and interactions in both recessive and dominant models. Genotypes homozygous and heterozygous for the major allele were used as the reference in the recessive model, and genotypes homozygous for the minor allele were used as the case. The dominant model comprised the homozygous genotype of the major allele as the reference and other two genotypes as the case. As we selected 4 SNPs, and each SNP was categorized into 2 groups (reference and case), the number of combination was 16 (2×2×2×2), including one combination with all references of 4 SNPs. To minimize multicollinearity, *p*-values for the interaction were calculated after each variable was centralized. A dose-dependent effect was assessed using the ORs according to the number of minor alleles as score variables (0–5). The number of alleles was counted as zero with a homozygous genotype for the major allele; one with a heterozygous genotype; and 2 with a homozygous genotype for the minor allele. The reference group was categorized as those with 0–3 minor alleles because of the small number of subjects. High HDL-C levels in the logistic regression model were defined as a level ≥62 mg/dL at the subject median in men and women. ORs and their 95% confidence intervals (CIs) were estimated after adjusting for age (continuous), sex, and regions (site 1 to site 6).

*P*-values less than 0.05 were considered statistically significant. We applied the Bonferroni correction in the analysis of SNPs to decrease the potential of an alpha error by multiple hypothesis testing. *P*-values less than 0.0038, 0.0033, or 0.010 were considered nominally significant in the regression analysis of the 13 SNPs; the 15 combinations for the selected 4 SNPs; and the reference and 5 categories for the number of alleles, respectively. Statistical calculations were performed using the Stata Version 10 software (Stata Corp, College Station, TX). Genotypes with distributions from the Hardy–Weinberg equilibrium (HWE) were assessed using the Chi-square test with the “genhwi” command in Stata.

## Results

The mean age and HDL-C values varied by region in both men and women (Table 1); the mean age ranged between 48.6 and 61.1 years in men and 46.4 and 60.2 years in women. The range of HDL-C values was between 55.0 and 64.7 mg/dL in men and 63.7 and 75.0 mg/dL in women. *P*-values for both the mean age and HDL-C values, determined using one-way ANOVA between the regions, were statistically significant by sex. The same degree of geographical variation in HDL-C values was not always observed between men and women.

**Table 1:** Geographical distribution of mean age and HDL-cholesterol values in the study subjects according to sex.

	Men					Women				
	N	Age (years)		HDL-C (mg/dL)		N	Age (years)		HDL-C (mg/dL)	
		Mean (SD)	*P* a	Mean (SD)	*P* a		Mean (SD)	*P* a	Mean (SD)	*P* a
Amami	193	55.5 (8.1)		58.3 (13.3)		276	54.7 (7.7)		63.7 (12.4)	
Saga	212	58.5 (7.9)		55.3 (14.2)		297	56.4 (7.9)		64.1 (13.9)	
Fukuoka	161	61.1 (5.6)		55.0 (16.1)		215	60.2 (5.5)		66.1 (15.9)	
Kyoto and Tokushima	178	48.6 (9.3)		59.0 (16.0)		53	46.4 (7.4)		67.5 (13.4)	
Takashima	154	59.2 (8.8)		60.5 (16.6)		322	56.3 (9.7)		67.7 (15.0)	
Okazaki	250	53.0 (8.3)		64.7 (17.8)		212	52.2 (9.0)		75.0 (16.0)	
Shizuoka	387	59.0 (8.8)		58.2 (15.3)		140	55.8 (8.4)		72.5 (16.2)	
Total	1,535	56.0 (9.1)	*<0.001*	58.9 (15.9)	*<0.001*	1,515	55.8 (8.6)	*<0.001*	67.5 (15.2)	*<0.001*

SD, standard deviation.

a) *P*-values were determined using one-way ANOVA between regions.

The mean age did not differ according to HDL-C levels in men and was slightly lower in women with high HDL-C (Table 2). The BMI averages and levels of LDL-C and triglycerides tended to be lower with high HDL-C in both men and women; however, the level of total cholesterol was positively correlated with the level of HDL-C. The level of METs in daily activity was positively correlated with the level of HDL-C in men. Proportionately more male smokers had low HDL-C, and more current drinkers, both men and women, had high HDL-C. Neither increasing nor decreasing trends were observed for the subjects who reported frequent intake of egg, meat, and fish.

**Table 2:** Characteristics of clinical lipid profiles and lifestyles in the study subjects according to sex.

	Men				Women			
	HDL-C (mg/dl)			*P*	HDL-C (mg/dl)			*P*
	<51.0	51.0–62.9	≥63.0		<60.0	60.0–72.9	≥73.0	
	(N = 516)	(N = 479)	(N = 540)		(N = 482)	(N = 501)	(N = 532)	
Age (years)	55.4	56.4	56.4	*0.344* a	57.3	55.4	54.8	*<0.001* a
BMI (kg/m^2^)	24.8	23.9	22.6	*<0.001* a	24.2	22.9	21.5	*<0.001* a
Total cholesterol (mg/dL)	199.4	205.3	212.0	*<0.001* a	214.5	220.0	222.4	*0.001* a
LDL-cholesterol (mg/dL)	121.3	123.1	116.2	*<0.001* a	134.9	133.1	122.6	*<0.001* a
HDL-cholesterol (mg/dL)	43.1	56.5	76.0	*<0.001* a	51.1	65.9	83.9	*<0.001* a
Triglyceride (mg/dL)	188.2	133.5	99.8	*<0.001* a	148.3	104.3	79.4	*<0.001* a
Daily activity (METs/day)	10.8	12.0	13.4	*<0.001* a	11.7	10.9	12.3	*0.134* a
Habitual exercise (METs/day)	0.46	0.57	0.58	*0.063* a	0.48	0.44	0.50	*0.434* a
Lifestyle (%)								
Current smoking	38.6	32.8	19.1	*<0.001* b	7.1	5.6	5.5	*0.503* b
Current drinking (≥3 times/week)	50.8	62.3	76.1	*<0.001* b	10.1	13.5	21.0	*<0.001* b
Egg intake (≥3 times/week)	54.8	59.1	57.8	*0.380* b	61.8	65.5	66.2	*0.308* b
Meat intake (≥3 times/week)	27.9	26.1	28.5	*0.672* b	39.6	40.5	43.2	*0.473* b
Fish intake (≥3 times/week)	51.4	54.5	55.0	*0.444* b	66.4	63.3	64.3	*0.582* b

Results are expressed as mean or as percentage; BMI, body mass index; LDL, low density lipoprotein; HDL, high density lipoprotein, METs; metabolic equivalents.

a) Trend test.

b) Chi square test.

The distribution of the minor allele frequency (MAF) from the selected 13 SNPs in the 4 genes is listed in Table 3. The MAF was distributed from 0.068 of *APOA1* rs12718465 to 0.495 of *CETP* rs5882. The genotype frequencies were all in HWE, with the exception of *CETP* (rs3764261; p<0.001). The geographical variation in the MAF of the *CETP* rs3764261 allele ranged from 0.173 to 0.337 depending on the region. Genotype frequencies in the Shizuoka and Okazaki regions were not in HWE, but the frequencies of the other 6 regions were in HWE. We also compared the MAF between the studied 3,050 subjects and 1,469 subjects who were excluded from analysis due to various reasons to evaluate the selection bias for excluded subjects. The genotype frequencies of all 13 SNPs among excluded 1,469 subjects were in HWE.

**Table 3:** Distribution of minor allele frequency in the presented 13 single nucleotide polymorphisms in 4 genes (n = 3050).

*Gene*	rs Number	Alias	Allele	MAF
*ABCA1*	rs2422493	−565C>T	C>T	0.408
	rs2230808	R1587K	G>A	0.399
	rs1800976	−273G>C	G>C	0.409
	rs2066718	V771M	G>A	0.075
	rs2740483	−17C>G	C>G	0.295
	rs2066715	V825I	G>A	0.361
*APOA1*	rs12718465	A61T	C>T	0.068
*CETP*	rs708272	Taq1B	C>T	0.398
	rs3764261	G/T	G>T	0.272
	rs5882	Ile405Val	A>G	0.495
	rs1800775	−629C>A	A>C	0.445
*SR-B1*	rs3782287	A>G	G>A	0.244
	rs5888	A350A	C>T	0.220

MAF, minor allele frequency.

We evaluated the effect of genetic and environmental factors on high HDL-C levels using the general linear models. The SNPs in *ABCA1* rs2422493, rs1800976, and rs2740483, and *CETP* rs708272, rs3764261, and rs1800775 were related to high HDL-C levels in model 1 after adjusting for age and sex (Table 4). SNPs (rs708272 and rs3764261) in the *CETP* gene were still statistically significant in model 2 after the Bonferroni correction (*p* = 0.003 and *p*<0.001, respectively), and the coefficient value (2.93 mg/dL/allele) for the *CETP* rs3764261 polymorphism was the highest among all the SNPs. Current drinking and daily activity were also associated with high HDL-C levels in both model 1 and 2 after adjusting for age, sex, environmental factors, and SNPs. Current smoking was negatively correlated with HDL levels. Egg intake had a borderline association with HDL-C levels in model 2.

**Table 4:** Coefficients of genetic and environmental factors in the general linear model for HDL-cholesterol levels.

Factors	Categories	Model 1^a^			Model 2^b^		
		Coef.	(95%CI)	*P* c	Coef.	(95%CI)	*P* c
Genetic factors^d^							
*ABCA1* rs2422493	C>T	1.20	(0.43–1.98)	*0.002*	0.49^e^	(−0.36 to 1.34)	*0.260*
*ABCA1* rs2230808	G>A	0.14	(−0.64 to 0.92)	*0.728*	0.44	(−0.32 to 1.2)	*0.255*
*ABCA1* rs1800976	G>C	1.21	(0.44–1.99)	*0.002*	0.47	(−0.38 to 1.32)	*0.279*
*ABCA1* rs2066718	G>A	−0.01	(−1.53 to 1.51)	*0.992*	−0.38	(−1.84 to 1.08)	*0.611*
*ABCA1* rs2740483	C>G	1.38	(0.54–2.22)	*0.001*	1.04	(0.12–1.96)	*0.027*
*ABCA1* rs2066715	G>A	0.56	(−0.23 to 1.36)	*0.166*	0.41	(−0.38 to 1.2)	*0.310*
*APOA1* rs12718465	C>T	2.13	(0.47–3.78)	*0.012*	1.80	(0.26–3.34)	*0.022*
*CETP* rs708272	C>T	2.99	(2.21–3.76)	*<0.001*	1.88	(0.63–3.14)	*0.003*
*CETP* rs3764261	G>T	4.11	(3.25–4.96)	*<0.001*	2.93	(1.90–3.97)	*<0.001*
*CETP* rs5882	A>G	0.58	(−0.17 to 1.32)	*0.129*	−0.76	(−1.64 to 0.12)	*0.091*
*CETP* rs1800775	A>C	1.74	(0.97–2.51)	*<0.001*	−0.23	(−1.34 to 0.88)	*0.689*
*SR-B1* rs3782287	G>A	0.34	(−0.55 to 1.23)	*0.456*	−0.05	(−0.91 to 0.82)	*0.914*
*SR-B1* rs5888	C>T	1.18	(0.26–2.10)	*0.012*	0.62	(−0.28 to 1.52)	*0.180*
Environmental factors							
Current smoking	(never, current)	−5.92	(−7.4 to −4.44)	*<0.001*	−7.00	(−8.42 to −5.59)	*<0.001*
Current drinking	(non, current)	7.32	(6.03–8.62)	*<0.001*	6.89	(5.68–8.09)	*<0.001*
Daily activity	(METs)	0.07	(0.03–0.11)	*0.001*	0.05	(0.01–0.09)	*0.027*
Habitual exercise	(METs)	1.20	(0.37–2.03)	*0.004*	0.61	(−0.16 to 1.38)	*0.122*
Egg intake	(1–8)	0.45	(0.03–0.87)	*0.034*	0.35	(−0.04–0.74)	*0.080*
Meat intake	(1–8)	0.20	(−0.39 to 0.8)	*0.503*	0.02	(−0.53–0.58)	*0.935*
Fish intake	(1–8)	0.35	(−0.17 to 0.88)	*0.189*	0.35	(−0.15–0.85)	*0.169*
Coef., coefficient.							

a) Adjusted for age (continuous), sex, and regions (site1 to site6).

b) Adjusted for age (continuous); sex; regions (site1 to site6); smoking; drinking; daily activity (metabolic equivalents: METs); habitual exercise (METs); intake of egg, meat, and fish; BMI; and SNPs except *ABCA1* rs2422493.

c) *P*-values less than 0.0038 were considered nominally significant for 13 SNPs after applied Bonferroni correction.

d) Score variables (1–3) were used according to number of reference alleles.

e) Adjusted for age (continuous); sex; regions (site1 to site6); smoking; drinking; daily activity (METs); habitual exercise (METs); intake of egg, meat, and fish; BMI; and SNPs except *ABCA1* rs1800976.

**Figure 3:**
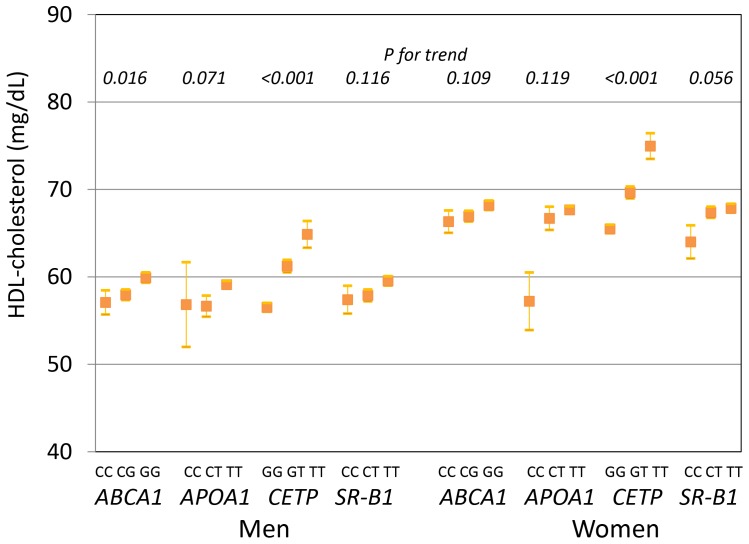
Comparison of HDL-C levels according to each genotype by sex and SNP. *P*-value was tested by the trend test.

To evaluate the gene-gene combination effect and interaction, we selected one SNP for each of the 4 genes (*ABCA1* rs2740483, *APOA1* rs12718465, *CETP* rs3764261, and *SR-B1* rs5888) that showed relatively stronger associations, with the lowest p-value and the highest coefficient for each gene in Table 4. At first, HDL-C levels were compared according to each genotype by sex and SNP. An apparent additive trend was observed in the *CETP* rs3764261 polymorphism in both men and women, and those with the *CETP TT* genotype had the highest HDL-C levels among men and women, respectively (Figure 3). The other 3 SNPs seemed to have either threshold or additive effects, although the increasing trend was not statistically significant.

The ORs for the high levels of HDL-C in combination with the selected 4 SNPs suggested a gene-gene combination effect in the recessive model considering the homozygote and heterozygote genotypes as the reference. Increased ORs were observed in every combination with *CETP* rs3764261, and the combination was statistically significant after the Bonferroni correction (Table 5). The combinations of 3 SNPs (*APOA1* rs12718465, *CETP* rs3764261, and *SR-B1* rs5888) and 4 SNPs (adding *ABCA1* rs2740483) had higher ORs (5.06, 2.72–9.39; and 4.74, 2.01–11.18, respectively) than any other combinations. The OR values in these combinations seemed to be higher than those obtained after multiplying each OR value, but their 95% CIs had a wide range. The association in the combination of the SNPs other than with *CETP* was not apparent. Gene-gene interactions between the 4 SNPs were not observed. We also identified the ORs in the dominant model by using only the homozygote genotype as the reference, but the ORs in many combinations were omitted from the calculation because of small number of references available, especially for *APOA1* (data not shown). We also performed the same analysis after excluding the regions not present in HWE, i.e., Shizuoka and Okazaki; excluding these regions from the analysis gave similar results as earlier (data not shown in Table). Increased ORs were also observed in every combination with *CETP* rs3764261, and the combination was statistically significant (p<0.0033). The combinations of 3 SNPs (*APOA1* rs12718465, *CETP* rs3764261, and *SR-B1* rs5888) and 4 SNPs (adding *ABCA1* rs2740483) had higher ORs (4.94, 2.13–11.43; and 4.52, 1.43–14.31, respectively) than any other combinations, too. The association in the combination of the SNPs other than with *CETP* was not apparent, except the combinations of 2 SNPs (*ABCA1* rs2740483 and *SR-B1* rs5888; 2.89, 1.53–5.39).

**Table 5:** Odds ratios (ORs) and 95% confidence intervals (CIs) for high HDL-cholesterol with the combination of selected single nucleotide polymorphisms (SNPs) and their interactions.

SNPs				Case/ref.	Combination			Interaction
					ORs^a^	(95% CI)	*P* b	*P* b
AB				1503/1547	1.18	(1.02–1.37)	*0.030*	*-*
	AP			2728/321	1.13	(0.88–1.43)	*0.336*	*-*
		CE		226/2823	2.38	(1.76–3.23)	*<0.001*	*-*
			SR	1820/1230	1.18	(1.01–1.37)	*0.035*	*-*
AB	+AP			1343/161	1.37	(0.97–1.93)	*0.072*	*0.737*
AB		+CE		104/1425	2.54	(1.62–3.98)	*<0.001*	*0.602*
AB			+SR	912/639	1.36	(1.10–1.68)	*0.005*	*0.198*
	AP	+CE		207/302	3.30	(2.14–5.09)	*<0.001*	*0.203*
	AP		+SR	1639/141	1.48	(1.03–2.12)	*0.035*	*0.374*
		CE	+SR	143/1147	3.00	(2.01–4.48)	*<0.001*	*0.225*
AB	+AP	+CE		96/150	3.75	(2.02–6.96)	*<0.001*	*0.207*
AB	+AP		+SR	819/74	1.81	(1.09–3.00)	*0.022*	*0.783*
AB		+CE	+SR	65/595	2.79	(1.54–5.03)	*0.001*	*0.325*
	AP	+CE	+SR	129/136	5.06	(2.72–9.39)	*<0.001*	*0.698*
AB	+AP	+CE	+SR	60/72	4.74	(2.01–11.18)	*<0.001*	*0.100*

Ref., reference; AB, *ABCA1* rs2740483 (CC and CG); AP, *APOA1* rs12718465 (CC and CT); CE, *CETP* rs3764261 (GG and GT); SR, *SR-B1* rs5888 (CC and CT).

a) Adjusted for age (continuous), sex and regions (site1-site6).

b) *P*-values less than 0.0033 were considered nominally significant after applied Bonferroni correction.

A dose-response effect on gene-gene interaction was also assessed. The ORs increased according to the number of alleles, and the dose-responsive trend was statistically significant (*p<*0.001; Table 6). We also conducted the same analysis after excluding the regions not present in HWE, i.e., Shizuoka and Okazaki. Excluding these regions from the analysis gave similar results as earlier, and the OR with 8 alleles was 6.01 (2.19–16.48) with significant dose-responsive trend (*p<*0.001; data not shown in Table).

**Table 6:** Odds ratios (ORs) and 95% confidence intervals (CIs) for high HDL-cholesterol with the number of alleles of selected single nucleotide polymorphisms.

Number of alleles^a^	N (%)	ORs^b^	(95% CI)	*P* c
0–3	171 (5.6)	1.00		
4	526 (17.3)	1.11	(0.77–1.61)	*0.580*
5	942 (30.9)	1.62	(1.14–2.30)	*0.007*
6	981 (32.2)	1.78	(1.25–2.53)	*0.001*
7	368 (12.1)	2.97	(1.98–4.45)	*<0.001*
8	60 (2.0)	5.48	(2.61–11.50)	*<0.001*
*P for trend* b		*<0.001*		

a) *ABCA1* rs2740483 (C = 0, G = 1); *APOA1* rs12718465 (C = 0, T = 1), *CETP* rs3764261 (G = 0, T = 1) and *SR-B1* rs5888 (C = 0, T = 1).

b) Adjusted for age (continuous), sex and regions (site1-site6).

c) *P*-values less than 0.01 were considered nominally significant after applied Bonferroni correction.

## Discussion

The present study investigated the interaction between gene polymorphisms in *ABCA1*, *APOA1*, *SR-B1*, and *CETP* for serum HDL-C in a cross-sectional study. We found a multiplier effect with *CETP* rs3764261 and the 3 other SNPs, with an increasing trend according to the number of alleles for serum HDL-C. To our knowledge, this is the second study to observe a gene-gene interaction for HDL-C [21] and the first study of its kind among the Asian population.

### Environmental Factors

The present study identified an association between environmental factors and HDL-C levels among the Japanese study population before investigating the gene-gene interactions. We observed a negative relationship between smoking and HDL-C levels. Previous studies were in concordance with this relationship and showed a potential influence on CETP activity [10], [28]. The relationship between alcohol consumption and increased HDL-C levels has been reported previously [6]. Alcohol consumption increases the expression of *ABCA1* [29] and the concentration of APOA1 levels [6] in peripheral blood and decreases the CETP activity [30]. The present study also revealed a positive relationship between alcohol consumption and HDL-C levels. Several interventional studies have reported a positive association between habitual exercise and HDL-C levels [7]. Exercise increases HDL-C levels in a population that already has normal levels of HDL-C but causes no or limited increase in subjects with already low HDL-C levels [31], [32]. Exercise also decreases the volume of fatty tissue and influences HDL-C levels [32], [33]. We observed increased HDL-C levels with daily activity.

Several reports have revealed a positive association between HDL-C levels and the consumption of fish and vegetable oil containing high concentrations of n-3 polyunsaturated fatty acids [9], [34]. An animal study found that the consumption of corn oil results in increased transport rates of HDL protein, as well as increased binding of HDL to liver membranes [35]. However, the results analyzing the association between fish consumption and increased HDL-C levels are inconsistent [36]. The present study also revealed no association between fish consumption and increased HDL-C levels. The present questionnaire did not include information about the types of fish species that were consumed, although different fish species, such as fatty fish, may have a different effect on cholesterol levels.

### Genetic Factors

Previous studies have shown that SNPs in the RCT system are responsible for varying levels of HDL-C [11]–[17]. The results of the present study also showed a significant relationship between the studied SNPs and serum HDL-C levels, except for *LCAT*, which had a lower MAF. The present results showed the highest coefficient for *CETP rs3764261*, which has also been observed in previous genome-wide screenings in the Japanese population and in the US EMR-linked biobanks [14], [21], [37]. A study that evaluated the Metabochip in African Americans reported an association between HDL-C and rs12740374 and rs17231520 in *CETP* [38]. We also observed a modest correlation with *ABCA1* rs2740483, and *APOA1* rs12718465. These SNPs are thought to play a partial role in influencing HDL-C levels because the combination with *CETP* rs3764261 shows a multiplier effect. The increasing trend with the number of alleles for serum HDL-C also supports the role of these SNPs, suggesting a dose-dependence on these alleles. The absence of an apparent interaction between these SNPs suggests that they may independently influence HDL-C levels. The previous study on gene-gene interaction using the US EMR-linked biobanks also adjusted for environmental factors such as BMI and smoking status [21]. The present study independently identified the effects of these SNPs after adjusting for a larger number of potential environmental factors in the general linear model.

The genotype frequency of *CETP* rs3764261 was not in HWE. Geographical differences in the allele frequency of the *CETP* rs3764261 polymorphism was observed in 2 regions (Okazaki and Shizuoka), and the genotype frequency was not in HWE in these regions. The deviation from HWE in these selected regions may potentially indicate population stratification rather than genotyping errors, because genotype frequencies of *CETP* polymorphisms in the 6 other regions were in HWE, and all SNPs were genotyped in the same laboratory at the same time. Okazaki and Shizuoka are located at the mainland of Japan, and their subjects were recruited from health checkup examinees of general population. Some selection bias may have influenced the genotype frequency of *CETP* polymorphisms, but its detail is unclear. We included the region in the analysis to control for regional CETP rs3764261 heterogeneity.

This study has several limitations, which need to be discussed. First, because this study was cross-sectional, the effects of diseases and medications on the results were not accounted for in our study. Therefore, we established strict exclusion criteria to remove these effects. Second, the lifestyle information obtained through the questionnaire includes a potential misclassification. To minimize this, we re-checked the questionnaire by employing trained interviewers. Third, geographic variation in SNPs in the Japanese population, including our study regions, has been previously reported [24], [39], [40]. Therefore, we included the region in question in our analysis to control for regional heterogeneity. Fourth, we did not analyze the fractions of HDL-C levels. Atheroprotective and non-atheroprotective HDL particles have different effects on coronary heart disease risk; therefore, fractional analysis of HDL-C levels is warranted in future studies. Fifth, bioinformaics on these genes and SNPs, and the function of SNPs are partially used to compute the allele dosage. We selected candidate SNPs of the genes in the RCT system by referring to previous reports that partially used bioinformatics and/or SNP functions, in order to compute allele dosage. Then, we selected one SNP from each gene in the RCT system after estimating their effects. Sixth, the potential of an alpha error needs to be considered, because the number of the present subjects was relatively large and multiple comparisons were made. Therefore, we applied Bonferroni correction. On the other hand, reducing an alpha error for null association increases a beta error for those associations that are not null. Several researchers mentioned that no adjustments are needed for multiple comparisons, although extended debate did not achieve consensus [41], [42]. Some results of smaller ORs in the present study become statistical significance, if Bonferroni correction was not applied, which did not influence the conclusion. Further biological relevance will be useful to support the present results.

In conclusion, the present study confirmed that smoking and drinking habits, daily activity, and polymorphisms in the *CETP* gene are associated with HDL-C levels. We found a multiplier effect from the *CETP* rs3764261 polymorphism in combination with *ABCA1*, *APOA1*, and *SR-B1* polymorphisms and a dose-dependency according to the number of alleles, although no gene-gene interaction was apparent.
